# Benefits of virtual reality based cognitive rehabilitation through simulated activities of daily living: a randomized controlled trial with stroke patients

**DOI:** 10.1186/s12984-016-0204-z

**Published:** 2016-11-02

**Authors:** Ana Lúcia Faria, Andreia Andrade, Luísa Soares, Sergi Bermúdez i Badia

**Affiliations:** 1 Madeira Interactive Technologies Institute, Funchal, Madeira Portugal; 2 Faculdade de Psicologia e de Ciências da Educação da Universidade de Coimbra, Coimbra, Portugal; 3 Universidade da Madeira, Funchal, Madeira Portugal

**Keywords:** Cognitive rehabilitation, Virtual reality, Ecological validity, Stroke

## Abstract

**Background:**

Stroke is one of the most common causes of acquired disability, leaving numerous adults with cognitive and motor impairments, and affecting patients’ capability to live independently. There is substancial evidence on post-stroke cognitive rehabilitation benefits, but its implementation is generally limited by the use of paper-and-pencil methods, insufficient personalization, and suboptimal intensity. Virtual reality tools have shown potential for improving cognitive rehabilitation by supporting carefully personalized, ecologically valid tasks through accessible technologies. Notwithstanding important progress in VR-based cognitive rehabilitation systems, specially with Activities of Daily Living (ADL’s) simulations, there is still a need of more clinical trials for its validation. In this work we present a one-month randomized controlled trial with 18 stroke in and outpatients from two rehabilitation units: 9 performing a VR-based intervention and 9 performing conventional rehabilitation.

**Methods:**

The VR-based intervention involved a virtual simulation of a city – Reh@City - where memory, attention, visuo-spatial abilities and executive functions tasks are integrated in the performance of several daily routines. The intervention had levels of difficulty progression through a method of fading cues. There was a pre and post-intervention assessment in both groups with the Addenbrooke Cognitive Examination (primary outcome) and the Trail Making Test A and B, Picture Arrangement from WAIS III and Stroke Impact Scale 3.0 (secondary outcomes).

**Results:**

A within groups analysis revealed significant improvements in global cognitive functioning, attention, memory, visuo-spatial abilities, executive functions, emotion and overall recovery in the VR group. The control group only improved in self-reported memory and social participation. A between groups analysis, showed significantly greater improvements in global cognitive functioning, attention and executive functions when comparing VR to conventional therapy.

**Conclusions:**

Our results suggest that cognitive rehabilitation through the Reh@City, an ecologically valid VR system for the training of ADL’s, has more impact than conventional methods.

**Trial registration:**

This trial was not registered because it is a small sample study that evaluates the clinical validity of a prototype virtual reality system.

## Background

In most countries, stroke is among most common causes of death and one of the main causes of acquired adult disability [1]. Because most patients with stroke survive the initial illness, the greatest impact is usually caused by the long term consequences for patients and their families [2]. It is estimated that 33 to 42 % of stroke survivors require assistance for daily living activities three to six months post stroke, and of these, 36 % continue to be disabled five years later [3, 4]. Although remarkable developments have been made in the medical treatment of stroke, it continues to heavily rely on rehabilitation interventions. In addition to motor disabilities, more than 40 % of stroke survivors are left with cognitive impairment after the event and almost two thirds are affected by mild cognitive impairment, and therefore are at risk of developing dementia [5]. Besides having a direct influence on the quality of life of patients and their caregivers, cognitive impairment after stroke is also associated with higher mortality [6] and greater rates of institutionalization [7]. Cognition is important for overall recovery since its impairment reduces a person’s ability to plan and initiate self-directed activities, to solve problems, to sustain and divide attention, to memorize information and to understand task instructions. It has been shown that recovery of cognitive function of stroke patients in inpatient rehabilitation is directly related to their level of participation in rehabilitation activities [8]. Thus, reducing the impact of post stroke cognitive impairment through appropriate rehabilitation programs is an essential goal.

Current cognitive rehabilitation practice tends to be directed towards isolated cognitive domains including attention (focusing, shifting, dividing or sustaining), executive functions (planning, inhibition, control), visuo-spatial ability (visual search, drawing, construction), memory (recall and recognition of visual and verbal information) and language (expressive and receptive) [9]. Although there is evidence on the efficacy of current methods [10], an important concern is how effectively the improvements of these abilities that are trained separately generalize, leading to sustained improvement in everyday functioning [11, 12]. When we consider the cognitive domains required for activities of daily living (ADL’s) such as a successful meal preparation – the patient must define a menu, identify the needed ingredients, write a shopping list, organize the time for shopping and preparing the meal – we acknowledge that multiple dimensions of cognition are engaged and, thereby, suggesting that need to be rehabilitated as a whole as opposed to independently [13]. Unfortunately, there is insufficient evidence to determine if and how the ecological validity of current cognitive rehabilitation methods impacts recovery [14, 15].

Current cognitive rehabilitation methodologies suffer other limitations besides the generalization of improvements to functional activities, social participation and life satisfaction. For instance, it is known that an intensive and individualized training is preferable [16]. Personalized rehabilitation involves an assessment of each patient’s impairments, a definition of attainable goals for improvement, an intervention to assist in the achievement of goals and, finally, a reassessment to measure improvements [2]. However, in-depth patient assessment is expensive and time consuming, and currently impracticable due to the scarcity of professionals and resources, resulting in a suboptimal intensity, personalization and duration of rehabilitation interventions [17]. Further, although there is growing evidence that patients may achieve improvements on functional tasks even many months after having a stroke [18], most rehabilitation therapies are only guaranteed within three to 6 months post stroke [19]. Additionally, a James Lind Alliance study [20] interviewed 799 chronic stroke patients who reported that cognitive problems had not been addressed appropriately, especially when compared with mobility, confirming that it is essential to find adaptable and accessible tools that can be used frequently and intensively by patients at the clinic or at home after discharge, in order to maximize rehabilitation outcomes. Caregivers and health professionals were also interviewed and indicated that investigating ways to improve cognition after stroke should be a research priority [21].

Virtual Reality (VR) and interactive technologies have emerged as a valuable approach in stroke rehabilitation by providing the opportunity to practice cognitive and motor activities that are not or cannot be usually practiced within the clinical environment, such as training attention abilities in street crossing situations [22], executive functions by visiting a supermarket [23], or performing simulations of real-life scenarios and activities in urban virtual environments [24, 25]. Yet, the advantages of VR to address stroke impairments go beyond ecological validity of training, with a growing body of evidence especially in the motor rehabilitation domain [26]. Virtual environments are designed to be more enjoyable than conventional rehabilitation methods. The introduction of gaming elements and immediate feedback on performance enhance motivation, thereby encouraging higher numbers of repetitions [27]. Additionally, it enables the systematic presentation of stimulus and challenges in a hierarchical fashion, which can be varied from simple to complex upon success [28], making it progressively challenging according to patients abilities. Further, when stroke survivors suffer of hemiparesis in their dominant arm, this interferes with their ability to perform paper-and-pencil tasks, which in turn may impede cognitive training. Thus, another central advantage of VR is the possibility to be integrated with accessible interfaces such as adapted joysticks, natural user interfaces or robotic systems [29].

Despite important scientific and engineering activity in VR based systems for cognitive and motor rehabilitation, the majority of studies to date have evaluated interventions that were designed to address motor impairments. According to the most recent Cochrane review [26], there are only few randomized controlled studies that include cognitive rehabilitation and/or cognition assessment. Kim and colleagues [30] performed a study with USN patients, where 12 experimental group patients received computer-based cognitive rehabilitation, including IREX system® (Vivid group, Toronto, Canada), and 12 control group patients received only computer-based cognitive rehabilitation with ComCog® (Maxmedica Inc., Seoul, Korea). Their results suggested that VR training might be a beneficial therapeutic technique on USN in stroke patients. Kim and colleagues [31] also investigated the effect of VR on the recovery of cognitive impairment in 28 stroke patients by comparing VR training with the IREX system® to computer-based cognitive rehabilitation with ComCog®. Results showed significant improvements in both groups, with the VR group having greater improvements in the attention domain. A study from Chirivella and colleagues [32] had 12 stroke patients in a stroke rehabilitation program using Neuro@Home, a cognitive and motor software-based rehabilitation platform. After an intervention of 8 weeks with 60 min sessions focused in attention, working memory, executive functions and visual perception training, patients showed significant improvements in attention, memory or executive functions. More recently and, in a more ADL’s simulation perspective, Gamito and colleagues [33] tested the effectiveness of a VR application for neuropsychological rehabilitation in a group of 20 stroke patients. Results showed significant improvements in attention and memory functions in the intervention group, but not in the control group, not subject to any intervention. Also in an ADL’s perspective, a pilot study from Rand and colleagues [34] explored the potential of a virtual supermarket (V-Mall) with 4 stroke patients. The intervention entailed ten 60-min sessions and was focused on improving multitasking while the participant was engaged in a virtual shopping task. Their main results support V-Mall potential as an effective tool for the rehabilitation of post stroke multitasking deficits during the performance of daily tasks. Most of these VR-based interventions do not address cognitive deficits in an integrative manner [30, 32, 33], or are not ecologically valid [30, 31]. The ADL’s simulation systems may represent a better real-world transfer rehabilitation, however, these systems lack difficulty customization [33, 34]. The AGATHE project developed a tool to suppress this demand, offering patients customized rehabilitation sessions through simulated ADL’s [25], however there are no efficacy clinical trials with this tool. Overall, we can conclude that results are encouraging but further research is needed, especially to clarify if VR, and more concretely training through the simulation of activities of daily living, is equivalent or more effective than conventional cognitive training [26].

In this paper we present a one-month clinical randomized controlled trial with 18 stroke patients: nine performing a VR-based intervention and nine performing a conventional intervention. The VR-based intervention involves a virtual simulation of a city – the Reh@City – where several activities of daily living are trained. Reh@City enables an integrative and personalized cognitive rehabilitation process, targeting several cognitive domains such as memory, attention, executive functions and visuo-spatial abilities in a more ecologically valid approach. Additionally, Reh@City makes the interaction with the virtual world accesible through its interface, and the complexity of the scenarios is adapted to the patients’ profile.

## Methods

### Participants

The selection of participants took place at the Nélio Mendonça and João Almada Hospitals (Madeira Health Service, Portugal). In total, we selected 18 out and inpatients, based on the following inclusion criteria: no hemi-spatial neglect as assessed by the clinicians with the Line Bisection test [35]; capacity to be seated; ability to read and write; minimum cognitive function as assessed by the Mini-Mental State Examination (MMSE) ≥ 15 [36]; and motivation to participate in the study. The Token Test [37] was used to identify and exclude patients with moderate or severe language comprehension deficits. The study was approved by the Madeira Health Service Ethical Committee (reference number: 47/2013) and all the patients gave informed consent previous to participation.

### Protocol

**Fig. 1:**
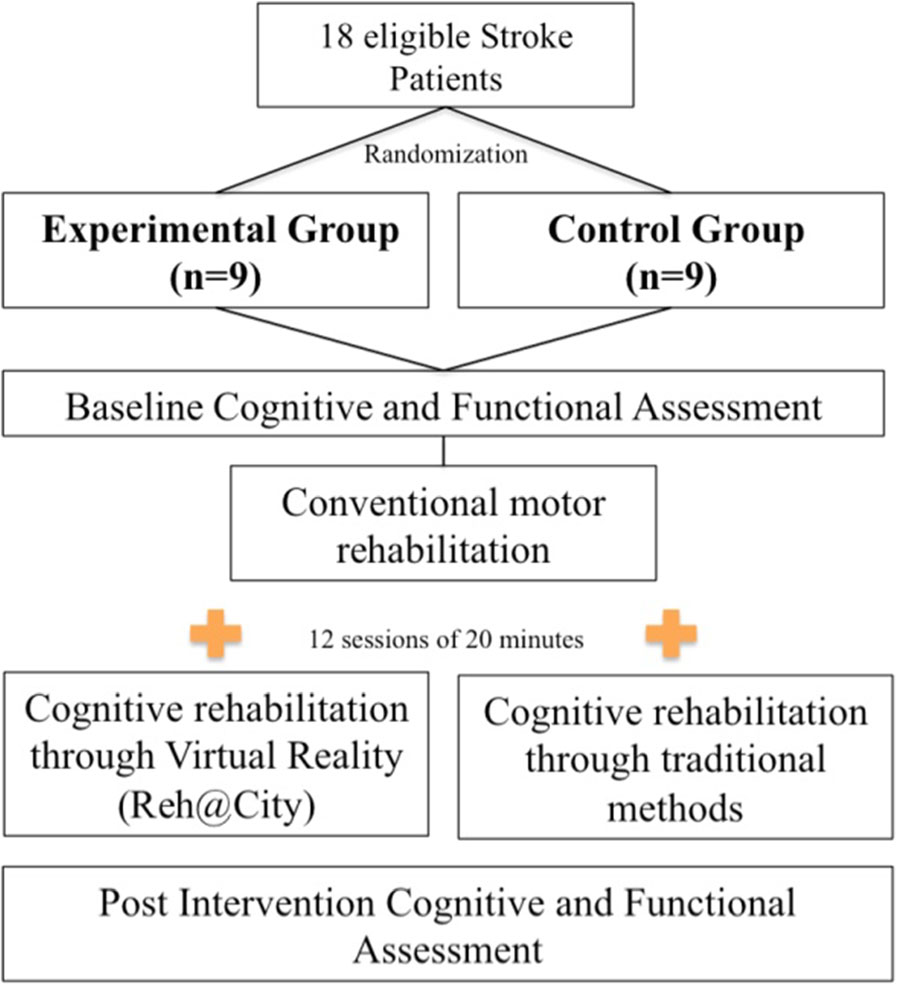
Protocol of the intervention

The 18 patients were randomly assigned to two different conditions: nine to the experimental group and nine to the control group (Fig. 1), by a researcher not involved in the collection of the data, using the Research Randomizer, a free web-based service that offers instant random sampling and random assignment [38]. Both groups underwent a twelve-session intervention, of 20 min each session, distributed from 4 to 6 weeks. Patients assigned to the experimental group used, during the training sessions under the supervision of a psychologist, a VR-based simulation of ADL’s, the Reh@City. The control group intervention involved time-matched cognitive training. Ideally, these participants should have performed the same simulated ADL’s in the real-world environment, as previously done in similar studies [39]. However, in addition to the logistics that could not be supported in this study (insurance and transportation outside the clinical environment), in this clinical population motor impairments would interfere with the tasks accomplishments and unsuccessful actions could be a result of motor instead of cognitive deficits. For this reason, and consistent with the current cognitive rehabilitation exercises at the study hospitals, patients performed puzzles, calculus, problem resolution and shape sorting involving the training of executive functions, visuo-spatial abilities, attention and memory, under the supervision of their occupational therapist.

### Simulation of ADL’s with the Reh@City

Paper and pencil tasks allow a very specific intervention in one or several cognitive domains but they lack ecological validity. In an attempt to address this limitation, our VR-based cognitive intervention consisted of a simulation of a city – Reh@City: a three-dimensional environment with streets, sidewalks, commercial buildings, parks and moving cars [40]. Because we are dealing with patients of generally older age and low computer literacy, the city was designed to have only square or rectangular building blocks and regular street intersections. This arrangement helps in memorizing the number of turns to get to a destination, and allows a more precise control of task difficulty.

**Fig. 2:**
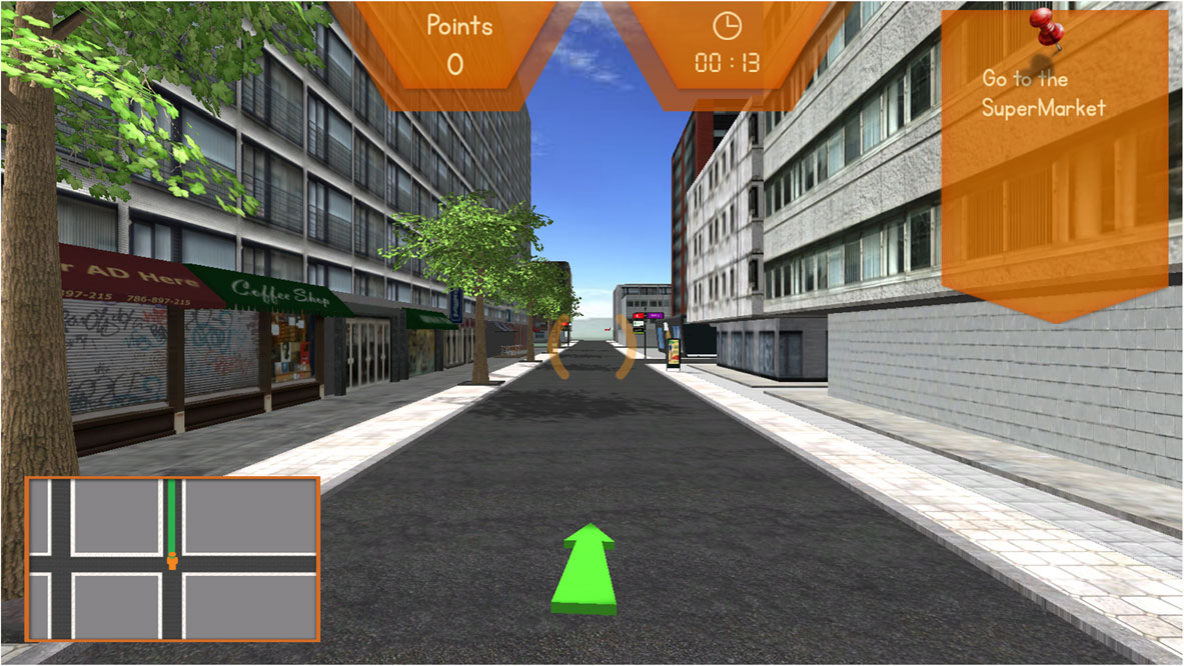
Three-dimensional street view of Reh@City. In a first-person navigation, users are given goal instructions supported with a mini-map indicating the optimal path (*green line and arrow*). Time and point counters are used to provide feedback on performance

Reh@City provides an integrative cognitive training experience where patients are required to accomplish some common ADL’s in four frequently visited places: a supermarket, a post office, a bank, and a pharmacy. To help the patient relate the VR tasks to the real world, these places display billboards and products of real spaces and trademarks commonly found in Portugal. When a task is given, the goal’s optimal path is displayed on a general map highlighted in green. The Reh@City can be configured to provide a mini-map in the lower half of the screen and/or a guidance arrow (Fig. 2), which allows increasing, or decreasing the visuo-spatial orientation demands involved in the navigation task. If needed, the patient can press a help button to recall the task instructions and have access again to the task map. Visual feedback elements, such as time and point counters, are used to give feedback on the accomplishment of the task objectives as well as to reward successful actions (Fig. 2). Points are accumulated at each objective completion (+20) and at each intermediate task (+1), and points are subtracted (−1) whenever a mistake is performed or a help button is used.

Attention training tasks bridge traditional paper and pencil cancellation tasks (where patients need to cross out target elements among distractors) and real tasks (where target and distractors are embedded in a real 3D environment). The implementation of the supermarket, the pharmacy and the post-office enables full control over the elements that determine the difficulty of training (number of targets, number of distractors and spatial arrangement of the grid). The list of tasks located in the up-right screen corner supports the patient by displaying the current objective and recently completed objectives. By removing the list we require the patient to memorize the sequence of tasks to perform. Further, the Reh@City targets executive functions by defining objectives that the patient needs to accomplish by using problem resolution, planning and reasoning skills (Table 1).

**Table 1:** Description of the levels of progression and cognitive domains involved

Levels of progression		Cognitive domains
1	Simple instructions (e.g. “Go to the supermarket and buy two bottles of water”) with mini-map, arrow and list of tasks cues	Visuo-spatial orientation and attention
2	Simple instructions (e.g. “Go to the Pharmacy and buy one cream”) without cues	Visuo-spatial orientation, attention and memory
3	Complex instructions (e.g. “Go to the Post-office buy two stamps and pick up three packages”) with mini-map, arrow and list of tasks cues	Visuo-spatial orientation, attention and executive functions (reasoning and planning)
4	Complex instructions (e.g. “Go to the supermarket and buy one orange juice, two boxes of cereals and four breads”) without cues	Visuo-spatial orientation, attention, memory and executive functions (reasoning and planning)
5	Problem resolution instructions (e.g. “Pay the electricity bill”) with mini-map, arrow and list of tasks cues	Visuo-spatial orientation, attention and executive functions (problem resolution, reasoning and planning)
6	Problem resolution instructions (e.g. “Get some food for breakfast”) without cues	Visuo-spatial orientation, attention, memory and executive functions (problem resolution, reasoning and planning)

### Accessibility

**Fig. 3:**
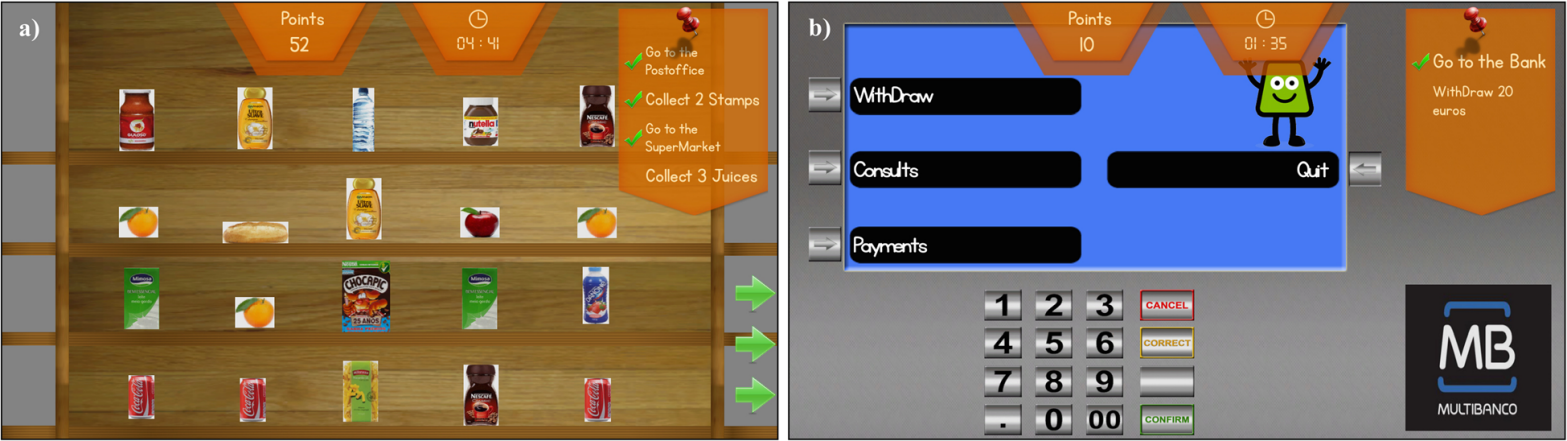
Examples of Reh@City ADL’s simulations. Representation in two dimensions of **a** supermarket shelves, and **b** a cash-machine

The navigation in the city is three-dimensional but the arrangement in the different locations, such as shelves and cash machine (Fig. 3a and b) are two dimensional to facilitate the selection of targets and to avoid motor difficulties in the interaction with hyper-realistic scenarios. Since most stroke patients have motor impairments, the navigation within the virtual environment was made through a joystick handle with only one button for “selection” and one for “help”. This simplified interface facilitates the learning process for those who never used a computer. A pilot study of the Reh@City prototype for a single session with 10 stroke patients [40] revealed a good level of usability (M = 77 %) as assessed through the System Usability Scale [41].

### Difficulty gradation and task personalization

Besides defining incrementally objectives with increased complexity (for instance “Go to the Supermarket and buy what is needed for breakfast”) (Table 1), we employed a method of fading cues: the Decreasing Assistance (DA) [42]. Following this methodology, in the first sessions the patient is immediately given all the cues available: mini-map; direction arrow and objectives list. The training continues with all the cues until correct performance is achieved on three consecutive sessions. On the following trial the cues supporting the well-succeeded actions are removed: if the patient easily navigates in the city, the direction arrow is removed; if the patient rapidly locates the places, the mini-map is removed; and if the patient correctly performs the objectives, the list is removed. If at any time the patient fails to produce the correct response, the cues are re-introduced until the performance is successful again.

### Reh@City implementation and setup

Reh@City was implemented using the Unity 3D game engine (Unity Technologies, San Francisco, USA). The experimental setup consisted of a desktop computer running Windows 7 (CPU: Intel core 2 duo, RAM: 4Gb) with a 24” LCD monitor. For the study an arcade type of joystick was used (Topway’s Digiusb Joystick Tp-usb670, China) with 2 customized colored buttons corresponding to the in-game actions “selection” and “help”.

### Neuropsychological assessment instruments

The same psychologist who supervised the experimental intervention assessed all participants for the trained cognitive domains before and after the interventions with a battery consisting of four neuropsychological instruments, with normative information available to indicate domain-specific deficits.

The primary outcome measure was the global cognitive functioning as assessed through the Addenbrooke Cognitive Examination (ACE) [43], which has good sensitivity (83 %) and specificity (73 %) for MCI after transient ischemic attack and stroke [44]. The ACE is built around the shell of the Mini-Mental State Examination (MMSE) [45] but assesses a wider range of cognitive functions. The application of the instrument takes 20 to 30 min and assesses attention and orientation, memory, verbal fluency, language and visuo-spatial abilities. Additionally, it provides the MMSE score, which was used as exclusion criteria for patients with severe cognitive deficits.

As secondary outcome measures, we had detailed attention and executive functioning assessments. To assess attention we used the Trail Making Test A and B (TMT A and B) [46], a very popular neuropsychological test that provides information on visual search, visual scanning, selective and divided attention, processing speed, mental flexibility, and also executive functioning. In part A, circles numbered from 1 to 25 needs to be connected in numerical order. In part B, numbers from 1 to 13 and letters from A to L need to be connected alternating numbers and letters in ascending order. To specifically assess **executive functions** we used the Picture Arrangement test from the Wechsler Adult Intelligence Scale III (WAIS III) [47]. This task consists of 11 sets of picture cards, presented in a standard mixed-up order, and the participant has to rearrange these to create a logical story within the specified time limit. It requires perceptual organization, sequencing, verbal comprehension and planning skills, as well as social knowledge.

Also, as a secondary outcome measure we had the subjective general health status, as assessed by the Stroke Impact Scale 3.0 (SIS 3.0), a self-reported questionnaire that functionally assesses 8 domains: motor strength, hand function, ADL’s, mobility (which are aggregated in the physical domain), communication, emotion, memory, and social participation [48]. The SIS 3.0 also includes patient’s subjective assessment on the perception of recovery since their stroke on a visual analog scale of 0 to 100, with 0 meaning no recovery and 100 meaning full recovery. Internal consistency and test-retest reliability of the SIS 3.0 domains ranges between 0.79 and 0.98 [49].

Both pre and post assessment moments had an approximate duration of 60 min. At the end of the VR-based intervention we additionally used the System Usability Scale (SUS) [41], to assess satisfaction and usability with the Reh@City system. Final scores for the SUS can range from 0 to 100, where higher scores indicate better usability: 90s is exceptional, 80s is good and 70s is acceptable [50]. The questionnaire is technology agnostic, making it flexible enough to assess a wide range of interface technologies.

### Data analysis

All statistical analyses were performed using SPSS software (version 20, SPSS Inc., Chicago IL, USA). As criterion for significance we used a α of 0.05. Normality of data was assessed with the Kolmogorov-Smirnov (KS) test. As some data were not normally distributed, nonparametric tests were used to evaluate the inter-group and intra-group differences. The Wilcoxon signed-rank test (W) was used to analyze the within group changes over time, while the two-tailed Mann-Whitney (MW) test was used to compare the between-group differences from baseline to the end of the study. No corrections for multiple comparisons were performed.

## Results

According to the Kolmogorov-Smirnov (KS) test, data were normally distributed in both groups for age (KS_Experimental_ = .156, *p* = .200; KS_Control_ = .196, *p* = .200) and in the control group for years of schooling (KS_Experimental_ = .394, *p* = .001; KS_Control_ = .267, *p* = .063). Data were not normally distributed for gender, lesion location and months post-stroke. No differences between groups were found with the Mann-Withney test (Table 2).

**Table 2:** Demographic characteristics (presented as Medians and IQR) of both groups and differences between groups (MW)

	Experimental (*n* = 9)	Control (*n* = 9)	*MW*	*p*
Age	58 (48–71)	53 (50.5–65.5)	35.000	.666
Gender	Female = 55.6 %; Male = 44.4 %	Female = 55.6 %; Male = 44.4 %	40.500	.100
Schooling	4 (4–10.5)	9 (4–9)	46.500	.605
Lesion location	Right = 55.6 %; Left = 44.4 %	Right = 55.6 %; Left = 44.4 %	36.000	.730
Months post-stroke	7 (4–49)	4 (3–11.5)	23.000	.136

Concerning the neuropsychological assessment measures at baseline, data were normally distributed in both groups for ACE (KS_Experimental_ = .218, *p* = .200; KS_Control_ = .185, *p* = .200) and only in the control group for the TMT A time (KS_Experimental_ = .390, *p* < .001; KS_Control_ = .169, *p* = .200) and the Picture Arrangement test (KS_Experimental_ = .371, *p* = .001; KS_Control_ = .240, *p* = .143). Data were also normally distributed in both groups for the subjective general health status for the memory (KS_Experimental_ = .227, *p* = .200; KS_Control_ = .122, *p* = .200), emotion (KS_Experimental_ = .254, *p* = .096; KS_Control_ = .147, *p* = .200), communication (KS_Experimental_ = .151, *p* = .200; KS_Control_ = .175, *p* = .200), ADL’s (KS_Experimental_ = .159, *p* = .200; KS_Control_ = .204, *p* = .200) an overall recovery (KS_Experimental_ = .269, *p* = .059; KS_Control_ = .264, *p* = .071) SIS dimensions. Social participation had a normal distribution only in the control group (KS_Experimental_ = .299, *p* = .020; KS_Control_ = .149, *p* = .200).

### Global cognitive functioning

Table 3 describes the global cognitive functioning, as assessed by the ACE, of both groups in the pre and post intervention assessments. A Wilcoxon test for within-groups differences revealed that only the experimental group presented significant statistical improvements between pre and post assessment moments in both ACE (W_(9)_ = 44.000, Z = −2.549, *p* = .011, *r* = .85) and MMSE (W_(9)_ = 34.000, Z = −2.246, *p* = .025, *r* = .75). Additionally, we also have found significant improvements in attention (W_(9)_ = 28.000, Z = −2.375, *p* = .018, *r* = .79), memory (W_(9)_ = 28.000, Z = −2.384, *p* = .017, *r* = .79) and visuo-spacial ability (W_(9)_ = 28.000, Z = −2.388, =.017, *r* = .80) domains only in the experimental group. Concerning the control group, the only significant change was a decline in verbal fluency (W_(9)_ = 2.500, Z = −2.209, *p* = .027, *r* = .74).

**Table 3:** ACE and MMSE scores (presented as Medians and IQR) pre and post intervention with within-groups (W) comparisons and pre to post-intervention difference with between-groups (MW) comparisons

	Experimental (*n* = 9)				Control (*n* = 9)					
	Pre	Post	*W*	*p*	Pre	Post	*W*	*p*	*MW*	*p*
ACE-Total	72 (61–75.5)	81 (68–86.5)	44.000	**.011**	66 (54.5–81)	69 (58–78)	24.000	.398	13.500	**.014**
MMSE	23 (20.5–26)	29 (25–29)	34.000	**.025**	23 (20.5–26)	26 (21–26.5)	28.500	.136	18.000	**.050**
ACE-Attention	15 (14–16.5)	18 (16.5–18)	28.000	**.018**	14 (12–16.5)	16 (12.5–17)	13.500	.518	17.500	**.040**
ACE-Memory	15 (13–18)	18 (15–21.5)	28.000	**.017**	18 (11–19.5)	18 (12.5–21)	11.000	.336	23.000	.136
ACE-Fluency	5 (2.5–6)	6 (4–7.5)	27.000	.196	6 (4–8)	5 (2.5–5.5)	2.500	**.027**	13.000	**.014**
ACE-Language	22 (21.5–23)	24 (21–26)	33.500	.191	19 (16–22)	21 (17–24.5)	22.000	.168	32.500	.489
ACE-Visuo-spatial	12 (7.5–14.5)	14 (13–15)	28.000	**.017**	12 (7.5–13.5)	14 (7–15.5)	16.000	.246	26.500	.222

*p* <.05 is indicated in bold

A Mann-Whitney test indicated that the experimental group improved, significantly more than the control group, in terms of general cognitive functioning, as assessed by ACE (U = 13.500, Z = −2.388, *p* = .014, *r* = .56) and MMSE (U = 18.000, Z = −1.996, *p* = .050, *r* = .47). The experimental group presented also significantly higher scores in the attention domain (U = 17.000, Z = −2.066, *p* = .040, *r* = .49). We also found significant differences between groups in the fluency task (U = 13.000, Z = −2.487, *p* = .014, *r* = .59) with improvements in the experimental group and decline in the control group. There were no differences between groups for memory (U = 23.000, Z = −1.578, *p* = 136, *r* = .37), language (U = 32.500, Z = −.713, *p* = 489, *r* = .17) and visuo-spatial (U = 26.500, Z = −1.263, *p* = .222, *r* = .30) domains.

### Attention

Table 4 describes the TMT A and TMT B performance for both groups, in terms of errors and completion time, pre and post intervention. No within group differences were identified by comparing the time to completion of the TMT A test in the experimental (W_(9)_ = 16.500, Z = −.711, *p* = .477, *r* = .24) and control (W_(9)_ = 17.500, Z = −1.153, *p* = .249, *r* = .38) groups, nor were there differences for the number of errors in the experimental (W_(9)_ = 1.000, Z = −1.089, *p* = .276, *r* = .36) and control (W_(9)_ = 5.000, *p* = −1.190, *p* = .234, *r* = .40) groups. Consistently for the TMT B, there were no differences for the time to completion in the experimental (W_(9)_ = 5.000, Z = −1.153, *p* = .249, *r* = .38) and the control (W_(9)_ = 3.000, Z = −1.572, *p* = .116, *r* = .52) groups, as well as differences in the number of errors in the experimental group (W_(9)_ = .000, Z = −1.890, *p* = .059, *r* = .63). However, we found differences in the control group (W_(9)_ = .000, Z = −2.060, *p* = .039, *r* = .69).

**Table 4:** TMT A, TMT B and Picture Arrangement scores (presented as Medians and IQR) pre and post intervention with within-groups (W) comparisons and pre to post-intervention difference with between-groups (MW) comparisons

	Experimental (*n* = 9)				Control (*n* = 9)					
	Pre	Post	*W*	*p*	Pre	Post	*W*	*p*	*MW*	*p*
A Time (seconds)	74 (53–160.5)	67 (60–110)	16.500	.477	120 (71.5–166)	97 (80.5–150)	17.500	.553	42.000	.931
A Errors	0 (0–3)	1 (0–1)	1.000	.276	1 (0–3)	1 (0–2)	5.000	.234	40.000	1
B Time (seconds)	360 (224–360)	240 (190–360)	5.000	.249	360 (334–360)	296 (226.5–360)	3.000	.116	43.500	.796
B Errors	4 (1.50–4)	3 (0–4)	.000	.059	4 (3–4)	3 (1.50–3.50)	.000	**.039**	35.500	.666
Pic. Arrangement	2 (0–2)	4 (1.50–6.50)	21.000	**.026**	2 (1–3.50)	2 (1–4)	2.000	.655	43.500	.063

*p* <.05 is indicated in bold

For the TMT A, both groups took less time to complete the post intervention test but with no significant differences between groups (U = 39.000, Z = −.132, *p* = .931, *r* = .03). For the TMT B, the experimental group took less time to completion when comparing to the control group, although this difference was not significant. There were no significant between group differences for the number of errors for both TMT A (U = 40.000, Z = .047, *p* = 1, *r* = .01) and TMT B (U = 35.500, Z = −.482, *p* = .666, *r* = .11).

### Executive functions

Table 4 describes the Picture Arrangement test performance for both groups pre and post intervention. In this executive functioning test, we have found significant differences within the experimental (W_(9)_ = 21.000, Z = −2.232, *p* = .026, *r* = .74) but not within the control (W_(9)_ = 2.000, Z = −.447, *p* = .655, *r* = .15) group. There was a tendency to significance for the experimental group to have better performance, when compared to the control, at the end of the intervention (U = 19.500, Z = −2.042, *p* = .063, *r* = .24).

### Subjective general health status

Table 5 describes the answers of both groups pre and post intervention to the SIS questionnaire. The SIS indicated that both groups perceived themselves as being better after the intervention. Improvements within the experimental group were significant for the physical domain (W_(9)_ = 43.000, Z = −2.431, *p* = .015, *r* = .81), namely strength (W_(9)_ = 28.000, Z = −2.388, *p* = .017, *r* = .80) and mobility (W_(9)_ = 36.000, Z = −2.527, *p* = .012, *r* = .84), memory (W_(9)_ = 40.000, Z = −2.081, *p* = .037, *r* = .69), emotion (W_(9)_ = 40.500, Z = −2.136, *p* = .033, *r* = .71), social participation (W_(9)_ = 34.000, Z = −2.240, *p* = .025, *r* = .75) and overall recovery (W_(9)_ = 28.000, Z = −2.401, *p* = .016, *r* = .80); but not for communication (W_(9)_ = 21.500, Z = −1.279, *p* = .201, *r* = .43), ADL’s (W_(9)_ = 38.000, Z = −1.840, *p* = .066, *r* = .61) and hand function (W_(9)_ = 23.500, Z = −1.614, *p* = .106, *r* = .54). The differences within the control group were significant for the physical dimension (W_(9)_ = 41.000, Z = −2.192, *p* = .028, *r* = .73), namely for the mobility (W_(9)_ = 26.000, Z = −2.028, *p* = .043, *r* = .68), memory (W_(9)_ = 36.000, Z = −2.524, *p* = .012, *r* = .84) and social participation (W_(9)_ = 36.000, Z = −2.521, *p* = .012, *r* = .84); but not for strength (W_(9)_ = 25.000, Z = −1.859, p = .063, *r* = .62), emotion (W_(9)_ = 30.000, Z = −1.682, *p* = .092, *r* = .56), communication (W_(9)_ = 20.000, Z = −1.014, *p* = .310, *r* = .34), ADL’s (W_(9)_ = 38.000, Z = −1.838, *p* = .066, *r* = .61), hand function (W_(9)_ = 18.000, Z = −1.594, *p* = .111, *r* = .53) and overall recovery (W_(9)_ = 30.500, Z = −1.763, *p* = .078, *r* = .59). There were no significant differences between groups in the strenght, mobility, hand function, ADL’s, memory, emotion, communication, social participation, and overall recovery dimensions of the SIS.

**Table 5:** SIS scores (presented as Medians and IQR) pre and post intervention with within-groups (W) comparisons and pre to post-intervention difference with between-groups (MW) comparisons

	Experimental (*n* = 9)				Control (*n* = 9)					
	Pre	Post	*W*	*p*	Pre	Post	*W*	*p*	*MW*	*p*
Physical	42.6 (35.5–56.9)	51.6 (37.7–71.7)	43.000	**.015**	39.4 (12.4–46.9)	38.1 (24.2–58.3)	41.000	**.028**	38.000	.863
Strength	50 (30–59.4)	62.5 (36.3–71.9)	28.000	**.017**	37.5 (12.5–53.1)	43.8 (25–62.5)	25.000	.063	40.000	.964
Memory	62.5 (45.3–82.8)	71.9 (53.1–86.6)	40.000	**.037**	56.3 (32.8–70.3)	62.5 (46.9–79.7)	36.000	**.012**	30.000	.387
Emotion	75 (55.5–84.7)	83.3 (75–87.4)	40.500	**.033**	58.3 (45.8–73.6)	66.67 ± 27.78	30.000	.092	50.500	.387
Communication	75 (60.7–91.1)	85.7 (62.5–94.6)	21.500	.200	67.9 (42.9–80.4)	67.9 (44.6–83.9)	20.000	.310	42.500	.863
Mobility	67.5 (42.5–74.9)	75 (51.3–86.3)	36.000	**.012**	40 (22.5–53.8)	52.5 (31.3–58.8)	26.000	**.043**	37.500	.790
Hand Function	15 (0–40)	40 (5–55)	23.500	.106	25 (0–30)	25 (0–45)	18.000	.111	37.000	.752
ADL’s	50 (37.5–80.2)	56.3 (49–86.5)	38.000	.066	43.8 (14.6–53.1)	45.8 (30.2–63.6)	38.000	.066	38.000	.863
Social	63.9 (29.2–72.3)	66.7 (53.5–83.3)	34.000	**.025**	36.1 (29.2–51.4)	50 (41.7–58.3)	36.000	**.012**	41.000	1
Recovery	50 (40–55)	70 (55–80)	28.000	**.016**	40 (40–55)	60 (45–75)	30.500	.078	31.500	.436

*p* <.05 is indicated in bold

### Usability

Although only 3 out of 9 participants from the experimental group had previous computer experience, there was a good acceptance of the system with no reported problems in the execution of the VR task. Observational information and subjective statements from the participants were consistent with the SUS scores, which reported good levels of usability and satisfaction for the Reh@City (Mdn = 80/100, IQR = 75–87.5).

## Discussion

In the past several VR systems have been developed for brain injury rehabilitation, some of which were developed but not field tested [24, 25] or have only gone through studies with a small number of participants and/or without control groups [23, 32, 51]. Most of the existing randomized controlled trials with VR-based cognitive rehabilitation, focus in specific cognitive domains, as memory [52, 53] and attention [33], or specific deficits, as USN [22, 30]. Instead, Reh@City was developed to target the rehabilitation of multiple cognitive domains simultaneously requiring the execution of daily routines in progressive levels of cognitive complexity. Our study, besides its limitations, is the first randomized controlled trial that shows evidence that VR-based cognitive rehabilitation in an ecologically valid context could be more effective than conventional training.

Comparing VR and control interventions, in terms of global cognitive functioning, as assessed with the ACE and the MMSE, only the experimental group improved significantly from pre to post-intervention. These significant improvements were also verified in the between-groups analysis. We have found significant improvements in attention, memory and visuo-spatial abilities for the experimental group. Attention and memory improvements are consistent with a study from Gamito and colleagues [33], which compared a VR-based intervention (ADL’s simulations targeting attention and memory) with conventional rehabilitation. The visuo-spatial improvements are consistent with Kim and colleagues [30] study, which compared a VR-based intervention with a computer-based intervention in USN. Considering executive functions, our control group had a significant decline in verbal fluency from pre to post intervention. The Picture Arrangement Test specifically assessed problem resolution and processing speed and its results revealed a pre to post intervention improvement only in the experimental group, which we consider a very promising result for further research.

The assessment of processing speed and attention with the TMT A and B revealed only a significant difference in the reduction of the number of errors, from pre to post intervention in the performance of the TMT B, in the control group. This result is not consistent with the other assessments and with previous studies, which found significant attention improvements, only in the experimental group [31]. The fact that this test is highly influenced by schooling [54] and that our sample had few years of education might explain the persistence of low performance in this test from pre to post assessment.

Besides cognition, we assessed the intervention’s impact in the multiple domains of health and life with the SIS 3.0. Self-reported data revealed that the experimental group improved significantly in the physical domain, namely strength and mobility, memory, emotion, social participation and overall recovery. Instead, the control group decreased in the physical domain and only improved in memory, mobility and social participation. Nevertheless, no differences between groups were identified. There are CID’s cut-offs for SIS 3.0 motor dimensions (strength = 9.2; ADL’s = 5.9; mobility = 4.5; hand function = 17.8) [55] and both groups’ improvements were clinically important for strength, ADL’s and mobility. These findings are especially relevant because our VR intervention targeted cognitive aspects but also improved the physical domain, more specifically motor strength, and the emotional condition of patients, as well as their own perception of overall recovery after stroke. Finally, the interaction with the our system was reported as very positive, with high levels of engagement and motivation, which is important to enhance adherence to treatment. The good usability and satisfaction scores obtained with the SUS confirmed these observations.

Despite the positive impact, some limitations of our study must be considered when interpreting the results. Concerning the sample, eighteen participants can be considered a small number, though it is comparable with previous similar interventions [31, 33]. In addition, there was heterogeneity between groups, especially related to time post-stroke. Although the experimental group was more chronic than the control, this difference was not statistically significant. The dosing of 4 h was of low intensity, and therefore might have not been sufficient to achieve greater or measurable improvements in both groups. Intervention duration of similar previous studies range from 6 to 18 h distributed in sessions of 30 to 60 min, 3 to 5 times a week [30–34]. Furthermore, the intervention was not blind since the same person performed the assessment and the intervention. Regarding the cognitive assessment, there might have been learning effects of the tools since none of them have parallel versions for multiple assessments. Yet, even if a learning effect existed, this would apply to both intervention and control groups and the comparison would still be valid. Nevertheless there are not established clinically important differences (CID’s) for the cognitive assessment tools, through the improvement scores from pre to post-intervention we can conclude that Reh@City, being it designed to address attention, memory, visuo-spatial abilities and executive functions, revealed to be more effective for cognitive rehabilitation than our control intervention. Although it would be relevant to have complementary information with a real-world assessment in a supermarket, pharmacy, post-office and bank, unfortunately this required logistics that could not be implemented for this study. In addition, the main objective was to clinically assess the impact of the Reh@City as a cognitive rehabilitation tool and not necessarily to assess the extent of transfer from VR to actual ADLs.

## Conclusions

This study examined the effectiveness of Reh@City in comparison to conventional methods. Overall, the results of this one-month longitudinal study have revealed that, cognitive rehabilitation through an ecologically valid VR system can have a larger impact than conventional methods. Reh@City showed similar functional impact as the conventional methods and larger improvements in general cognitive functioning. Our results contribute with new evidence and provide further understanding on the impact of using simulations of ADL’s in the rehabilitation of cognitive deficits. Nevertheless there is still a need of further research considering other clinical populations, larger sample sizes and more comparative studies. Hence, a comparison of an improved version of this VR system with a comprehensive paper-and-pencil cognitive training, using a greater number of patients is taking place.

## Abbreviations

ACE: Addenbrooke Cognitive Examination
ADL’s: Activities of daily living
DA: Decreasing Assistance
MMSE: Mini-Mental State Examination
SIS 3.0: Stroke Impact Scale 3.0
SUS: System Usability Scale
TMT A: Trail Making Test A
TMT B: Trail Making Test B
USN: Unilateral spatial neglect
VR: Virtual reality
WAIS III: Wechsler Adult Intelligence Scale III

## References

[1] World Health Organization. The top 10 causes of death. July 2013. 2015. Available at: who.int/mediacentre/factsheets/fs310/en/. [Accessed July 2015].

[2] Langhorne P, Bernhardt J, Kwakkel G (2011). Stroke rehabilitation. Lancet.

[3] Feigin VL, Barker-Collo S, McNaughton H, Brown P, Kerse N (2008). Long-term neuropsychological and functional outcomes in stroke survivors: current evidence and perspectives for new research. Int J Stroke.

[4] Brainin M, Norrving B, Sunnerhagen KS, Goldstein LB, Cramer SC, Donnan GA (2011). Poststroke chronic disease management: towards improved identification and interventions for poststroke spasticity-related complications. Int J Stroke.

[5] Madureira S, Guerreiro M, Ferro JM (2001). Dementia and cognitive impairment three months after stroke. Eur J Neurol.

[6] Rajan KB, Aggarwal NT, Wilson RS, Everson-Rose SA, Evans DA (2014). Association of cognitive functioning, incident stroke, and mortality in older adults. Stroke.

[7] Pasquini M, Leys D, Rousseaux M, Pasquier F, Hénon H (2007). Influence of cognitive impairment on the institutionalisation rate 3 years after a stroke. J Neurol Neurosurg Psychiatry.

[8] Skidmore ER, Whyte EM, Holm MB, Becker JT, Butters MA, Dew MA (2010). Cognitive and affective predictors of rehabilitation participation after stroke. Arch Phys Med Rehabil.

[9] Cumming TB, Marshall RS, Lazar RM (2013). Stroke, cognitive deficits, and rehabilitation: still an incomplete picture. Int J Stroke.

[10] Cicerone KD, Langenbahn DM, Braden C, Malec JF, Kalmar K, Fraas M (2011). Evidence-based cognitive rehabilitation: updated review of the literature from 2003 through 2008. Arch Phys Med Rehabil.

[11] Sohlberg MM, Mateer CA. Cognitive rehabilitation: An integrative neuropsychological approach. Guilford Press; 2001.

[12] Karbach J, Verhaeghen P (2014). Making working memory work: a meta-analysis of executive-control and working memory training in older adults. Psychol Sci.

[13] Wilson BA, Herbert CM, Shiel A. Behavioural approaches in neuropsychological rehabilitation: Optimising rehabilitation procedures. Psychology Press; 2004.

[14] van Heugten C, Gregório GW, Wade D (2012). Evidence-based cognitive rehabilitation after acquired brain injury: a systematic review of content of treatment. Neuropsychol Rehabil.

[15] Parsons TD. Neuropsychological Rehabilitation 3.0: State of the Science. In: Clinical Neuropsychology and Technology. Springer International Publishing; 2016. p. 113–32.

[16] Cicerone KD, Dahlberg C, Malec JF, Langenbahn DM, Felicetti T, Kneipp S (2005). Evidence-based cognitive rehabilitation: updated review of the literature from 1998 through 2002. Arch Phys Med Rehabil.

[17] Teasell RW, Foley NC, Salter KL, Jutai JW (2008). A blueprint for transforming stroke rehabilitation care in Canada: the case for change. Arch Phys Med Rehabil.

[18] Teasell RW, Murie Fernandez M, McIntyre A, Mehta S (2014). Rethinking the continuum of stroke rehabilitation. Arch Phys Med Rehabil.

[19] Aziz N (2010). Long-term rehabilitation after stroke: where do we go from here?. Rev Clin Gerontol.

[20] McKevitt C, Fudge N, Redfern J, Sheldenkar A, Crichton S, Wolfe C (2010). UK stroke survivor needs survey.

[21] Pollock A, St George B, Fenton M, Firkins L (2014). Top 10 research priorities relating to life after stroke – consensus from stroke survivors, caregivers, and health professionals. Int J Stroke.

[22] Navarro MD, Lloréns R, Noé E, Ferri J, Alcañiz M (2013). Validation of a low-cost virtual reality system for training street-crossing. A comparative study in healthy, neglected and non-neglected stroke individuals. Neuropsychol Rehabil.

[23] Kizony R, Korman M, Sinoff G, Klinger E, Josman N (2012). Using a virtual supermarket as a tool for training executive functions in people with mild cognitive impairment.

[24] Dores AR, Miranda MJ, Carvalho IP, Mendes L, Barbosa F, Coelho A (2012). Virtual City: neurocognitive rehabilitation of acquired brain injury. 2012 7th Iberian Conference on Information Systems and Technologies (CISTI).

[25] Klinger E, Kadri A, Sorita E, Le Guiet J-L, Coignard P, Fuchs P (2013). AGATHE: a tool for personalized rehabilitation of cognitive functions based on simulated activities of daily living. IRBM.

[26] Laver KE, George S, Thomas S, Deutsch JE, Crotty M. Virtual reality for stroke rehabilitation. The Cochrane Library; 2015.

[27] Laver K, George S, Thomas S, Deutsch JE, Crotty M (2012). Cochrane review: virtual reality for stroke rehabilitation. Eur J Phys Rehabil Med.

[28] Riva G. Virtual Reality in Neuro-psycho-physiology: Cognitive, Clinical and Methodological Issues in Assessment and Rehabilitation. IOS Press; 1997. 10175335

[29] Faria AL, Vourvopoulos A, Cameirão MS, Fernandes JC, Bermúdez i Badia S. An integrative virtual reality cognitive-motor intervention approach in stroke rehabilitation: a pilot study. In: 10th ICDVRAT, Gothenburg, Sweden: The University of Reading; Sept 2–4, 2014.

[30] Kim YM, Chun MH, Yun GJ, Song YJ, Young HE (2011). The effect of virtual reality training on unilateral spatial neglect in stroke patients. Ann Rehabil Med.

[31] Kim BR, Chun MH, Kim LS, Park JY (2011). Effect of virtual reality on cognition in stroke patients. Ann Rehabil Med.

[32] Chirivella J, del Barco A, Blasco S, Penades V, Mas G, Gagliardo P (2014). Neuro@ home [ii]: A software platform of clinically designed videogames designed for the cognitive rehabilitation of stroke patients. Brain Injury.

[33] Gamito P, Oliveira J, Coelho C, Morais D, Lopes P, Pacheco J (2015). Cognitive training on stroke patients via virtual reality-based serious games. Disabil Rehabil.

[34] Rand D, Weiss PLT, Katz N (2009). Training multitasking in a virtual supermarket: a novel intervention after stroke. Am J Occup Ther.

[35] Schenkenberg T, Bradford DC, Ajax ET (1980). Line bisection and unilateral visual neglect in patients with neurologic impairment. Neurology.

[36] Guerreiro M, Silva AP, Botelho MA, Leitão O, Castro-Caldas A, Garcia C (1994). Adaptação à população portuguesa da tradução do Mini Mental State Examination (MMSE). Revista Portuguesa de Neurologia.

[37] De Renzi A, Vignolo LA (1962). Token test: a sensitive test to detect receptive disturbances in aphasics. Brain.

[38] Research Randomizer [Internet] (2016). Research randomizer.

[39] Aubin G, Béliveau M-F, Klinger E (2015). An exploration of the ecological validity of the Virtual Action Planning–Supermarket (VAP-S) with people with schizophrenia. Neuropsychol Rehabil.

[40] Vourvopoulos A, Faria AL, Ponnam K, Bermúdez i Badia S. RehabCity: Design and Validation of a Cognitive Assessment and Rehabilitation Tool through Gamified Simulations of Activities of Daily Living. In: 11th International Conference on Advances in Computer Entertainment Technology. Funchal, Portugal: The University of Reading; 2014.

[41] Brooke J (1996). SUS-A quick and dirty usability scale. Usability Eval Ind.

[42] Riley GA, Heaton S (2000). Guidelines for the selection of a method of fading cues. Neuropsychol Rehabil.

[43] Mioshi E, Dawson K, Mitchell J, Arnold R, Hodges JR (2006). The Addenbrooke’s Cognitive Examination Revised (ACE-R): a brief cognitive test battery for dementia screening. Int J Geriatr Psychiatry.

[44] Pendlebury ST, Mariz J, Bull L, Mehta Z, Rothwell PM (2012). MoCA, ACE-R, and MMSE Versus the National Institute of Neurological Disorders and Stroke–Canadian Stroke Network Vascular Cognitive Impairment Harmonization Standards Neuropsychological Battery After TIA and Stroke. Stroke.

[45] Folstein MF, Folstein SE, McHugh PR (1975). “Mini-mental state”: a practical method for grading the cognitive state of patients for the clinician. J Psychiatr Res.

[46] Reitan RM (1958). Validity of the trail making test as an indicator of organic brain damage. Percept Mot Skills.

[47] Ryan JJ, Lopez SJ, Dorfman WI, Hersen M (2001). Wechsler adult intelligence scale-III. Understanding psychological assessment [internet].

[48] Duncan PW, Bode RK, Min Lai S, Perera S (2003). Rasch analysis of a new stroke-specific outcome scale: the Stroke Impact Scale. Arch Phys Med Rehabil.

[49] Vellone E, Savini S, Fida R, Dickson VV, Melkus GD, Carod-Artal FJ (2015). Psychometric evaluation of the stroke impact scale 3.0. Journal of Cardiovascular Nursing.

[50] Bangor A, Kortum PT, Miller JT (2008). An empirical evaluation of the system usability scale. Int J Hum Comput Interact.

[51] Jacoby M, Averbuch S, Sacher Y, Katz N, Weiss PL, Kizony R (2013). Effectiveness of executive functions training within a virtual supermarket for adults with traumatic brain injury: a pilot study. IEEE Trans Neural Syst Rehabil Eng.

[52] Optale G, Urgesi C, Busato V, Marin S, Piron L, Priftis K (2010). Controlling memory impairment in elderly adults using virtual reality memory training: a randomized controlled pilot study. Neurorehabil Neural Repair.

[53] Yip BC, Man DW (2013). Virtual reality-based prospective memory training program for people with acquired brain injury. Neurorehabilitation.

[54] Tombaugh TN (2004). Trail Making Test A and B: normative data stratified by age and education. Arch Clin Neuropsychol.

[55] Lin K, Fu T, Wu C, Wang Y, Liu J, Hsieh C (2010). Minimal detectable change and clinically important difference of the stroke impact scale in stroke patients. Neurorehabil Neural Repair.

